# nsink: An R package for flow path nitrogen removal estimation

**DOI:** 10.21105/joss.04039

**Published:** 2022-03-11

**Authors:** Jeffrey W. Hollister, Dorothy Q. Kellogg, Qian Lei-Parent, Emily Wilson, Cary Chadwick, David Dickson, Arthur Gold, Chester Arnold

**Affiliations:** 1 U. S. Environmental Protection Agency, Atlantic Coastal Environmental Sciences Division, Narragansett, RI 02882; 2 University of Rhode Island, Department of Natural Resources Science, Kingston, RI 02881; 3 University of Connecticut, Center for Land Use Education and Research, Storrs, CT 06268

## Abstract

The nsink package estimates cumulative nitrogen (N) removal along a specified flow path and is based on methodologies outlined in Kellogg et al. (2010). For a user-specified watershed (i.e., hydrologic unit code (HUC)), nsink downloads all required datasets from public datasets in the United States, prepares data for use, summarizes N removal along a flow path and creates several static maps. The results of an nsink analysis may be exported to standard geospatial files for use in other applications.

## Statement of need

Excess N delivery via surface water to downstream aquatic resources contributes to impaired water quality and impacts ecosystem services including harmful algal blooms (HABs) and hypoxia (Rabalais et al., 2002). Identifying landscape N sinks (i.e., areas where N is effectively removed from the aquatic system) and analyzing N delivery at the watershed scale is helpful to watershed managers, land use planners and conservation organizations. The theoretical underpinnings for identifying N sinks rely on decades of research and are explained in Kellogg et al. (2010).

Prior N-sink implementations were done case-by-case. Data acquisition and manipulation were mostly manual and took weeks to months to complete for a single 12-digit HUC. The effort required for the analysis limited it’s application as scaling beyond a few pilot studies was not feasible. The goal of nsink was to address this limitation and provide an open source solution that could be run on a single small watershed (e.g., 12-digit HUC) in minutes to hours with minimal manual input.

## The nsink package

### Package Installation

The nsink package is available from https://github.com/usepa/nsink and may be installed in R with the following:


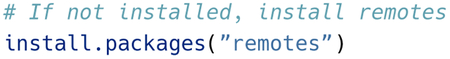



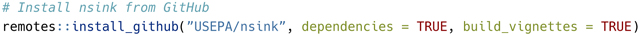


### Package Details

The nsink package is designed around the major steps in running an N-Sink analysis and includes functions for the following tasks:

1. Prepare for analysis
  - Get data
  - Prepare data for analysis
  - Calculate relative N removal layer for hydric soils, lakes and streams.
2. Run a point-based analysis
  - Calculate a flow path
  - Summarize relative N removal along a flow path
3. Run a HUC-based analysis
  - Develop static maps
  - Generate output datasets

#### Required Data

The ability to run an nsink analysis relies on several datasets for the conterminous United States. By limiting our approach to these national datasets we are ensuring scalability of nsink because the datasets will be available for most locations in the United States. The datasets that nsink uses are the National Hydrography Dataset Plus version 2 (NHDPlus), Soil Survey Geographic Database (SSURGO), the National Land Cover Dataset (NLCD) land cover, and the National Land Cover Dataset (NLCD) impervious surface (Jin et al., 2019; Moore et al., 2019; Soil Survey Staff, 2017). These datasets are all available via an Application Programming Interface (API) or via direct download.

#### Dependencies

The nsink package depends on several existing R packages to facilitate spatial data handling, data acquisition, data management, data analysis and data processing. These are detailed in Table 1.

#### Functionality

Currently, nsink provides 10 exported functions to facilitate a flow path analysis of relative N removal. The nsink repository (https://github.com/usepa/nsink) and R package documentation contain detailed documentation of each function. The pacakge also has a vignette that outlines a typical workflow for running an N-Sink analysis with the nsink package. Upon install, the vignette is accessed in R with vignette(“intro”, package = “nsink”).

## Figures and Tables

**Table 1.** R package dependencies for the nsink package

Package	Task	Citation
sf	Spatial Data Handling and Analysis	Pebesma (2018); Pebesma (2021b)
raster	Spatial Data Handling and Analysis	Hijmans (2021)
stars	Spatial Data Handling and Analysis	Pebesma (2021c)
fasterize	Spatial Data Handling and Analysis	Ross (2020)
lwgeom	Spatial Data Handling and Analysis	Pebesma (2021a)
gstat	Spatial Data Handling and Analysis	Pebesma (2004); Gräler et al. (2016); Pebesma & Graeler (2021)
sp	Spatial Data Handling and Analysis	Pebesma & Bivand (2005); Bivand et al. (2013); Pebesma & Bivand (2021)
units	Unit Transformations	Pebesma et al. (2016); Pebesma et al. (2021)
FedData	Data Acquisition	Bocinsky (2020)
httr	Data Acquisition	Wickham (2020)
dplyr	Data Management and Analysis	Wickham et al. (2021)
zoo	Data Management and Analysis	Zeileis & Grothendieck (2005); Zeileis et al. (2021)
igraph	Data Management and Analysis	Csardi & Nepusz (2006); Csardi et al. (2020)
readr	Data Management and Analysis	Wickham & Hester (2020)
foreign	Data Management and Analysis	R Core Team (2020)
rlang	Data Management and Analysis	Henry & Wickham (2021)
furrr	Parallel Processing	Vaughan & Dancho (2021)
future	Parallel Processing	Bengtsson (2021); Bengtsson (2020)

## References

[R1] Bengtsson H (2020). Future: Unified parallel and distributed processing in r for everyone. https://CRAN.R-project.org/package=future

[R2] Bengtsson H (2021). A Unifying Framework for Parallel and Distributed Processing in R using Futures. In The R Journal. 10.32614/RJ-2021-048

[R3] Bivand RS, Pebesma EJ, & Gomez-Rubio V (2013). Applied spatial data analysis with R, second edition. Springer, NY. 10.1007/978-1-4614-7618-4

[R4] Bocinsky RK (2020). FedData: Functions to automate downloading geospatial data available from several federated data sources. https://CRAN.R-project.org/package=FedData

[R5] Csardi G, & Nepusz T (2006). The igraph software package for complex network research. InterJournal, Complex Systems, 1695. https://igraph.org

[R6] Csardi G, Nepusz T, Horvat S, Traag V, & Zanini F (2020). Network analysis and visualization. https://CRAN.R-project.org/package=zoo 10.1371/journal.pone.0355108 PMC13436765 42550872

[R7] Gräler B, Pebesma EJ, & Heuvelink G (2016). Spatio-temporal interpolation using gstat. The R Journal, 8, 204–218. 10.32614/RJ-2016-014

[R8] Henry L, & Wickham H (2021). Rlang: Functions for base types and core r and ‘tidyverse’ features. https://CRAN.R-project.org/package=rlang

[R9] Hijmans RJ (2021). Raster: Geographic data analysis and modeling. https://CRAN.R-project.org/package=raster

[R10] Jin S, Homer C, Yang L, Danielson P, Dewitz J, Li C, Zhu Z, Xian G, & Howard D (2019). Overall methodology design for the united states national land cover database 2016 products. Remote Sensing, 11(24), 2971. 10.3390/rs11242971

[R11] Kellogg DQ, Gold AJ, Cox S, Addy K, & August PV (2010). A geospatial approach for assessing denitrification sinks within lower-order catchments. Ecological Engineering, 36(11), 1596–1606. 10.1016/j.ecoleng.2010.02.006

[R12] Moore RB, McKay LD, Rea AH, Bondelid TR, Price CV, Dewald TG, Johnston CM others. (2019). User’s guide for the national hydrography dataset plus (NHDPlus) high resolution. Open-File Report-US Geological Survey, 2019–1096. 10.3133/ofr20191096

[R13] Pebesma EJ (2004). Multivariable geostatistics in S: The gstat package. Computers & Geosciences, 30, 683–691. 10.1016/j.cageo.2004.03.012

[R14] Pebesma EJ (2018). Simple Features for R: Standardized Support for Spatial Vector Data. The R Journal, 10(1), 439–446. 10.32614/RJ-2018-009

[R15] Pebesma EJ (2021a). Lwgeom: Bindings to selected ‘liblwgeom’ functions for simple features. https://CRAN.R-project.org/package=lwgeom

[R16] Pebesma EJ (2021b). Simple features for r. https://CRAN.R-project.org/package=sf

[R17] Pebesma EJ (2021c). Stars: Spatiotemporal arrays, raster and vector data cubes. https://CRAN.R-project.org/package=stars

[R18] Pebesma EJ, & Bivand R (2021). Sp: Classes and methods for spatial data. https://CRAN.R-project.org/package=sp

[R19] Pebesma EJ, & Bivand RS (2005). Classes and methods for spatial data in R. R News, 5(2), 9–13. https://CRAN.R-project.org/doc/Rnews/

[R20] Pebesma EJ, & Graeler B (2021). Gstat: Spatial and spatio-temporal geostatistical modelling, prediction and simulation. https://CRAN.R-project.org/package=gstat

[R21] Pebesma EJ, Mailund T, & Hiebert J (2016). Measurement units in R. R Journal, 8(2), 486–494. 10.32614/RJ-2016-061

[R22] Pebesma EJ, Mailund T, Kalinowski T, & Ucar I (2021). Units: Spatiotemporal arrays, raster and vector data cubes. https://CRAN.R-project.org/package=units

[R23] R Core Team. (2020). Foreign: Read data stored by ‘minitab’, ‘s’, ‘SAS’, ‘SPSS’, ‘stata’, ‘systat’, ‘weka’, ‘dBase’, … https://CRAN.R-project.org/package=foreign

[R24] Rabalais NN, Turner RE, & Scavia D (2002). Beyond science into policy: Gulf of mexico hypoxia and the mississippi river: Nutrient policy development for the mississippi river watershed reflects the accumulated scientific evidence that the increase in nitrogen loading is the primary factor in the worsening of hypoxia in the northern gulf of mexico. BioScience, 52(2), 129–142. 10.1641/0006-3568(2002)052[0129:BSIPGO]2.0.CO;2

[R25] Ross N (2020). Fasterize: Fast polygon to raster conversion. https://CRAN.R-project.org/package=fasterize

[R26] Soil Survey Staff, U. (2017). Web soil survey.

[R27] Vaughan D, & Dancho M (2021). Furrr: Apply mapping functions in parallel using futures. https://CRAN.R-project.org/package=furrr

[R28] Wickham H (2020). Httr: Tools for working with URLs and HTTP. https://CRAN.R-project.org/package=httr

[R29] Wickham H, François R, Henry L, & Müller K (2021). Dplyr: A grammar of data manipulation. https://CRAN.R-project.org/package=dplyr

[R30] Wickham H, & Hester J (2020). Readr: Read rectangular text data. https://CRAN.R-project.org/package=readr

[R31] Zeileis A, Gorthendieck G, & Ryan JA (2021). Zoo: S3 infrastructure for regular and irregular time series (Z’s Ordered Observations). https://CRAN.R-project.org/package=zoo

[R32] Zeileis A, & Grothendieck G (2005). Zoo: S3 infrastructure for regular and irregular time series. Journal of Statistical Software, 14(6), 1–27. 10.18637/jss.v014.i06

